# Developmentally Programmed Division of Labor in the Aquatic Invader *Alternanthera philoxeroides* Under Homogeneous Soil Nutrients

**DOI:** 10.3389/fpls.2019.00485

**Published:** 2019-04-16

**Authors:** Dao-Guo Xi, Wen-Hua You, An-An Hu, Ping Huang, Dao-Lin Du

**Affiliations:** Institute of Environment and Ecology, College of the Environment and Safety Engineering, Jiangsu University, Zhenjiang, China

**Keywords:** alligator weed, biomass allocation, chlorophyll fluorescence, clonal growth, physiological integration

## Abstract

Clonal traits can contribute to plant invasiveness, but little is known about the roles of division of labor (a key clonal trait) in homogeneous habitats. The hypothesis tested is that clonal integration allows division of labor and increases the overall performance of an invasive clonal plant, especially under higher soil nutrients. Clonal fragment pairs of aquatic invader *Alternanthera philoxeroides* (each with four ramets and a stolon apex) were grown in two homogenous habitats with high or low soil nutrient supply, and with stolon connections being either severed (clonal integration prevented) or kept intact (clonal integration allowed). Results showed that stolon connection allowed the division of labor within the clonal fragment, with basal ramets specializing in acquisition of belowground resources and apical ramets specializing in acquisition of aboveground expansion. Moreover, the capacity for division of labor was greater, which brought the clonal fragments of *A. philoxeroides* stronger clonal propagation and better performance in high nutrient habitats than in low nutrient habitats. The results supported our hypotheses that the developmentally programmed division of labor may facilitate the clonal expansion of this aggressive invader in some homogeneous habitats with high resource availability.

## Introduction

Invasive plants have greatly threatened biodiversity, the environment and economic development worldwide (Mack et al., 2000; Vila et al., 2011; van Kleunen et al., 2015). While the mechanisms that contribute to the successful invasion of exotic plants remain unsolved (Alpert et al., 2000; Levine et al., 2003; Vila et al., 2011), an emerging pattern in plant invasion is that a great number of aggressive invaders are clonal plants (Kolar and Lodge, 2001; Liu et al., 2006; Xu et al., 2010; You et al., 2013; Keser et al., 2014; Roiloa et al., 2016). Recently, an increasing number of studies have pointed out that the clonal traits such as clonal integration and division of labor may contribute to the invasiveness of these invaders (Aguilera et al., 2010; Roiloa et al., 2010; Song et al., 2013; You et al., 2014a, 2016a; Roiloa et al., 2016; Wang Y.J. et al., 2017). However, so far, the knowledge of contribution of clonal traits on invasion success of these clonal invaders is still limited (Song et al., 2013; Keser et al., 2014; Wang Y.J. et al., 2017).

A key clonal trait is the capacity for division of labor (Hutchings and Wijesinghe, 1997; Stuefer, 1998), which is mediated by physiological integration and driven by the source–sink relationship (Roiloa and Retuerto, 2006; You et al., 2013). Due to physiological integration, when two essential resources are heterogeneously distributed (negatively correlated), the connected ramets can specialize to acquire the resource that is relatively more abundant within the clone to enhance the overall performance of the clone (Hutchings and Wijesinghe, 1997; Stuefer, 1998; Roiloa et al., 2007). Such specialization is called “division of labor,” which is environmentally induced (Stuefer, 1998; Roiloa et al., 2016). In comparison, when the clone consists of the ramets that are in different developmental stages, or differing in ability to acquire resources, the connected ramets within the clone may get different amounts of resources even with the same external resource supply (Dong et al., 2015). In this case, division of labor and physiological integration between ramets may still increase plant performance even in the homogeneous environments, with relatively older ramets specializing in taking up belowground resources and relatively younger ramets specializing in aboveground resources and spread (Stuefer, 1998; Roiloa et al., 2013). Ramet specialization in such homogeneous environments is termed “developmentally programmed division of labor” (Stuefer, 1998; Roiloa et al., 2013), which is inherent in clonal plants (Roiloa et al., 2013). Although division of labor is beneficial to clonal plants, the importance of ramet specialization for the invasiveness of clonal plants is still far from clear (Roiloa et al., 2016).

The essential resources for plants in many habitats are commonly heterogeneously distributed (Caldwell and Pearce, 2012), however, other natural and some anthropogenic habitats are relatively homogeneous (Dutilleul, 2011; Dong et al., 2015). For example, some shallow rivers and wetlands where water movement can homogenize the habitats, and many anthropogenic habitats such as irrigation ditches and crop lands could be homogeneous (Dong et al., 2015). These homogeneous habitats are expected to be easier to invade by exotic plants because of their relatively lower biodiversity than other natural habitats (Levine and D’Antonio, 1999; You et al., 2016a). However, very few studies have investigated the role of the division of labor in shaping the invasion success of plant invaders in homogeneous habitats (Roiloa et al., 2013; Wang P. et al., 2017). Recently, through a conceptual model, Dong et al. (2015) discovered that physiological integration may also increase the performance of a notorious plant invader when its connected ramets differ in ability to acquire resources in homogeneous environments with high resource supply. Based on this conceptual model, it can be predicted that clonal plants containing ramets that are in different developmental stages or differing in ability to acquire resources may have a greater capacity for the division of labor, and thereby benefit from physiological integration more in homogenous habitats with high resource supply than with low resource supply. Unfortunately, no previous study has tested this prediction.

Using clonal fragments of an amphibious clonal invader *Alternanthera philoxeroides*, we conducted a greenhouse experiment to test the above prediction. We grew ramet pairs of *A. philoxeroides* in two homogenous habitats, either with high soil nutrient supply or with low soil nutrient supply, with stolon connections either severed (clonal integration prevented) or kept intact (clonal integration allowed). Here, we tested the following hypotheses: (1) Stolon connection (clonal integration) allows division of labor between ramets of *A. philoxeroides* clonal fragments in homogenous habitats. We test if the division of labor happens for *A. philoxeroides* in homogeneous habitats in term of the biomass allocation (i.e., root mass to shoot mass ratio). Considering that the connected ramets of *A. philoxeroides* are in different developmental stages (i.e., basal ramets are relatively older and apical ramets are relatively younger), we expect that stolon connection will increase biomass allocation to roots of basal (older) ramets (specialization in the uptake of belowground resources). Meanwhile, we expect that stolon connection will increase biomass to shoots and enhance the photochemical efficiency (as indicators of the energy allocated to harvest aboveground resources) of apical (younger) ramets. (2) Clonal integration increases the overall performance of the clonal fragments. We predict higher photochemical efficiency, clonal propagation and biomass production, resulting in higher growth capacity and invasiveness in connected clonal fragments than severed ones. (3) The capacity for division of labor is stronger and its benefits to *A. philoxeroides* clonal fragment is greater when grown in high soil nutrient than in low soil nutrients. Based on the conceptual model presented by Dong et al. (2015), we expect a greater division of labor (i.e., basal ramets specializing in taking up belowground resources and apical ramets specializing in the uptake of aboveground resources), resulting in higher performance of clonal fragments grown in high soil nutrient than in low soil nutrients.

## Materials and Methods

### Plant Species

*Alternanthera philoxeroides* (Mart.) Griseb. (Amaranthaceae), commonly called “alligator weed,” is an amphibious, perennial clonal plant native to South America (Julien et al., 1995). It has caused serious environmental and economic problems both locally and globally (Julien et al., 1995; Gunasekera and Bonila, 2001). *A. philoxeroides* rarely produces seeds and propagates vegetatively by stems and root buds (Julien et al., 1995; Schooler, 2012). In its introduced regions, *A. philoxeroides* has invaded widely from aquatic to terrestrial habitats, such as irrigation ditches and crop lands where are relatively homogenous due to anthropogenic activities (Geng et al., 2007; Dong et al., 2015; You et al., 2016a). In China, the genetic diversity of *A. philoxeroides* is extremely low (Xu et al., 2003; Wang et al., 2005), and an increasing number of studies have demonstrated that physiological integration and the division of labor may determine its growth and spread (Wang et al., 2008; Yu et al., 2009; You et al., 2016b, 2018).

### Experimental Design

**FIGURE 1:**
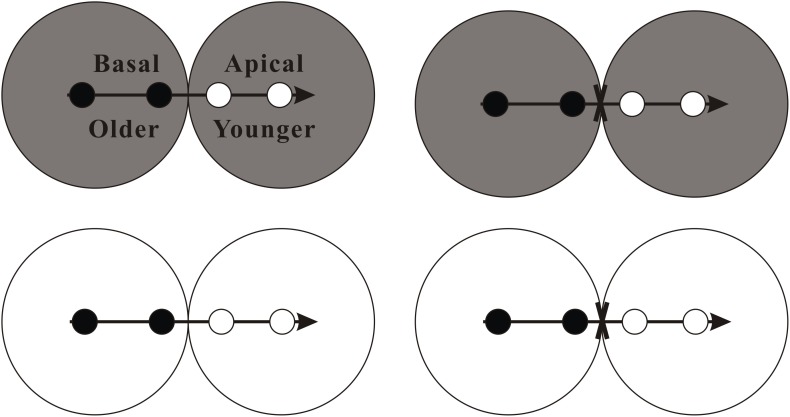
Schematic representation of the experimental design. There were four experimental treatments involving stolon connection and soil nutrient availability. Clonal fragments of the invasive plant *A. philoxeroides*, each consisting of two basal ramets (black circles) and two apical ramets (white circles) with a stolon apex (horizontal arrow), were grown either under high soil nutrient availability (gray) or under low soil nutrient availability (white), and with the stolon connections between basal and apical ramets were either intact or severed (fork). See text for additional explanation.

In mid-April 2016, we collected plant material of *A. philoxeroides* from the surrounding wetlands of Gonghu Bay in the Taihu Lake, Jiangsu province of China (N 31°25^′^–31°28^′^, E 120°15^′^–120°21^′^) and then propagated them in a greenhouse. In this experiment, we used 32 clonal fragments of *A. philoxeroides* in similar size (tip cuttings, 14.32 ± 0.16 cm in length, 0.37 ± 0.07 g in dry mass; means ± SE) as experimental material. Each clonal fragment contained four ramets with a stolon apex, which were divided into two parts: a “basal part” with two relatively old ramets (distal to the tip), an “apical part” with two relatively young ramets (close to the tip) and a stolon apex (see Figure 1). Within each clonal fragment, the basal part was placed in the basal pot, and the apical part was placed in the apical pot (Figure 1).

The experiment was conducted in a greenhouse in the Field Station of Jiangsu University, by a full factorial design with stolon connection (stolon connections were intact or severed) and soil nutrient (high nutrient or low nutrient) as fixed factors. For the stolon connection treatment, the connection between basal and apical ramets in each clonal fragment was either severed (clonal integration prevented) or kept intact (clonal integration allowed). After the two original ramets of both apical and basal part rooted, the stolon connections were cut halfway between the basal and apical ramets. There was no negative effect of the severing treatment observed during the experiment (immediate death or disease). New ramets for both basal and apical part produced during the experiment were not allowed to root. All the experimental pots (12 cm in upper diameter, 8.8 cm in bottom diameter and 10.8 cm tall) were filled with the mixture of clean river sand and green zeolite (water retention) at a volume of 3: 1. For high and low soil nutrient treatments, the experimental pots were mixed evenly with 2 g and 0.4 g of slow-release fertilizer (Osmocote^R^, N–P–K: 16–9–12) powder, respectively. Each treatment combination was replicated eight times (*n* = 8). During the experimental period, the mean light intensity was 1200–1500 μmol m^-2^ s^-1^ at noon and the mean air temperature was 25–28°C in the greenhouse. To mimic a natural wetland habitat condition, plants were watered regularly to keep the soil with an overlying water of 2 cm deep. The experiment was conducted for 9 weeks and ended on June 28, 2016.

### Measurements

The chlorophyll fluorescence was measured 3 days before the final harvest. According to the saturation pulse method (Schreiber et al., 1998; Maxwell and Johnson, 2000), after a more than 20 min’ dark adaptation, the minimum (*F*_0_) and the maximum (*F*_m_) fluorescence yield were measured on a healthy mature leaf of the second-youngest ramet by a portable chlorophyll fluorometer (PAM-2100, Walz, Effeltrich, Germany). (*F*_m_ – *F*_0_)/*F*_m_ was defined as the maximum quantum yield of PSII (*F*_v_/*F*_m_). Similarly, after an actinic light pulse of 120 μmol m^-2^ s^-1^ for 10 s, *F’*_m_ is measured as the maximal fluorescence yield reached in a pulse of saturating light, and *F*_t_ is the fluorescence yield of the leaf at that photosynthetic photon flux density. Then the effective quantum yield of PS II (Yield) was calculated as (*F’*_m_ – *F*_t_)/*F’*_m_ (Roiloa and Retuerto, 2006; You et al., 2013). The chlorophyll content index (the relative chlorophyll content) was also measured by a portable chlorophyll meter (TYS-A, TOP, Zhejiang, China). The leaves used for measuring the relative chlorophyll content were opposite to the leaves used for determination of *F*_v_/*F*_m_ and Yield.

Nine weeks after the beginning of the experiment, all the clonal fragments were harvested. The number of ramets (i.e., number of nodes) were counted, and the total stolon length (e.g., the sum of main stolon length and branch stolon length) were measured for both apical and basal ramets. Then, all the *A. philoxeroides* plants were separated into leaves, stolons, and roots, and each part was weighed after drying to constant weight at 70°C for 72 h.

### Statistical Analysis

Before the analysis, to meet the assumptions of normality and homoscedasticity, the data were log-transformed (the proportions were angular transformed) if necessary. The growth measurements (final biomass, total stolon length, and ramet number) for the apical part, the basal part and the whole clonal fragment, the physiological measurements (*F*_v_/*F*_m_, Yield and chlorophyll content index) and root mass/shoot mass ratios (hereafter denoted by R/S ratio) for the apical part and the basal part were analyzed by Two-way ANOVAs, using stolon connection and soil nutrient level as fixed factors. To examine differences between the treatments, we used Studentized Tukey’s HSD for multiple comparisons. Statistical significance was assigned at *P* < 0.05. All data analyses were performed by SPSS 18.0 (SPSS, Chicago, IL, United States).

## Results

### Biomass Allocation and Growth

**FIGURE 2:**
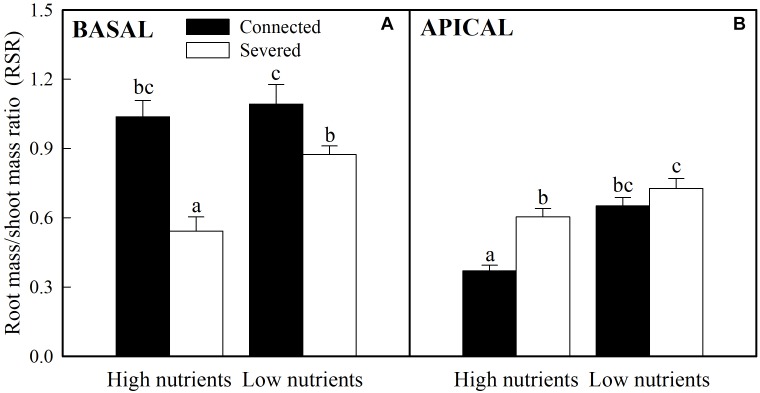
Effects of experimental treatments on the root to shoot mass ratio of basal **(A)** and apical **(B)** ramets of *A. philoxeroide*. The data indicate the means + SE (*n* = 8). The bars sharing the same letter are not significantly different at *P* = 0.05.

Soil nutrient, stolon connection and their interaction significantly affected biomass allocated to roots of both basal (older) and apical (younger) ramets, as determined by the R/S ratio (Table 1). Stolon connection greatly increased the proportion of biomass allocated to roots in basal ramets (Figure 2A) whereas decreased it in apical ramets (the effect of stolon connection on R/S ratio for apical ramets in low nutrient treatment was marginally significant, *P* = 0.09) (Figure 2B). Such effects of stolon connection on basal and apical ramets were significantly stronger when *A. philoxeroides* was grown in high soil nutrients than in low soil nutrients, as demonstrated by the significant effects of nutrient × stolon connection (Figure 2 and Table 1).

**Table 1:** Two-way ANOVA analyses for the effects of soil nutrient and stolon connection on the growth (final biomass, total stolon length, and total node number) and root mass to shoot mass ratio (R/S ratio) of *Alternanthera philoxeroides* for the basal part, apical part, and whole clonal fragment.

Dependent variable	Nutrient (N)	Connection (C)	N × C
**Basal**			
Final biomass	197.41^***^	14.21^**^	4.12^*^
Total stolon length	84.08^***^	22.74^***^	0.10
Total node number	65.98^***^	13.30^**^	0.15
R/S ratio	8.37^**^	31.74^**^	4.91^*^
**Apical**			
Final biomass	121.03^***^	25.62^***^	1.77
Total stolon length	92.96^***^	31.36^***^	4.94^*^
Total node number	57.05^***^	30.49^***^	4.80^*^
R/S ratio	27.63^***^	18.81^***^	7.88^**^
**Whole fragment**			
Final biomass	242.66^***^	5.20^*^	3.98^*^
Total stolon length	220.48^*^	7.02^*^	4.80^*^
Total node number	166.19^***^	8.55^**^	4.01^*^
d.f.	1.28	1.28	1.28


*Values give *F*; significant *P*-values are indicated by ^∗^. ^∗^*P* < 0.05, ^∗∗^*P* < 0.01, and ^∗∗∗^*P* < 0.001.*

**FIGURE 3:**
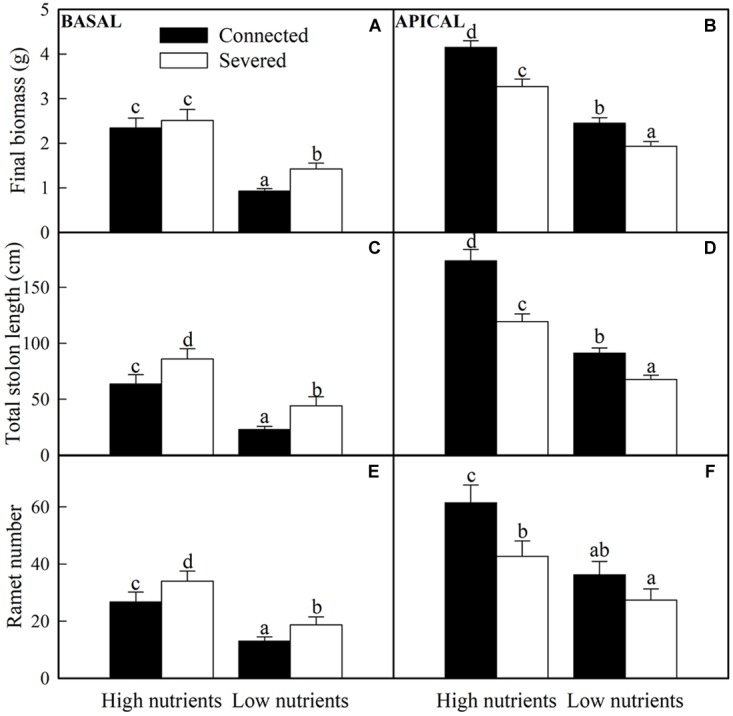
Effects of experimental treatments on the final biomass, total stolon length and ramet number of basal **(A,C,E)** and apical **(B,D,F)** ramets of *A. philoxeroide*. The data indicate the means + SE (*n* = 8). The bars sharing the same letter are not significantly different at *P* = 0.05.

All the growth measures (e.g., final biomass, ramet number, and total stolon length) of basal and apical ramets were significantly affected by soil nutrient and stolon connection (Table 1). The final biomass of basal ramets, total solon length and ramet number of apical ramets were also significantly affected by soil nutrient × stolon connection (Table 1). High nutrient treatment greatly promoted the growth measures of both basal and apical ramets (Figure 3). Stolon connection significantly decreased the growth of basal ramets (except the final biomass of *A. philoxeroides* grown in high soil nutrient) (Figure 3A,C,E), whereas the growth of apical ramets were greatly improved by stolon connection, and such positive effects on apical ramets were stronger when *A. philoxeroides* was grown in high soil nutrient than in low soil nutrient, as demonstrated by the significant effects of nutrient × stolon connection for total stolon length and ramet number (Figure 3B,D,F and Table 1).

**FIGURE 4:**
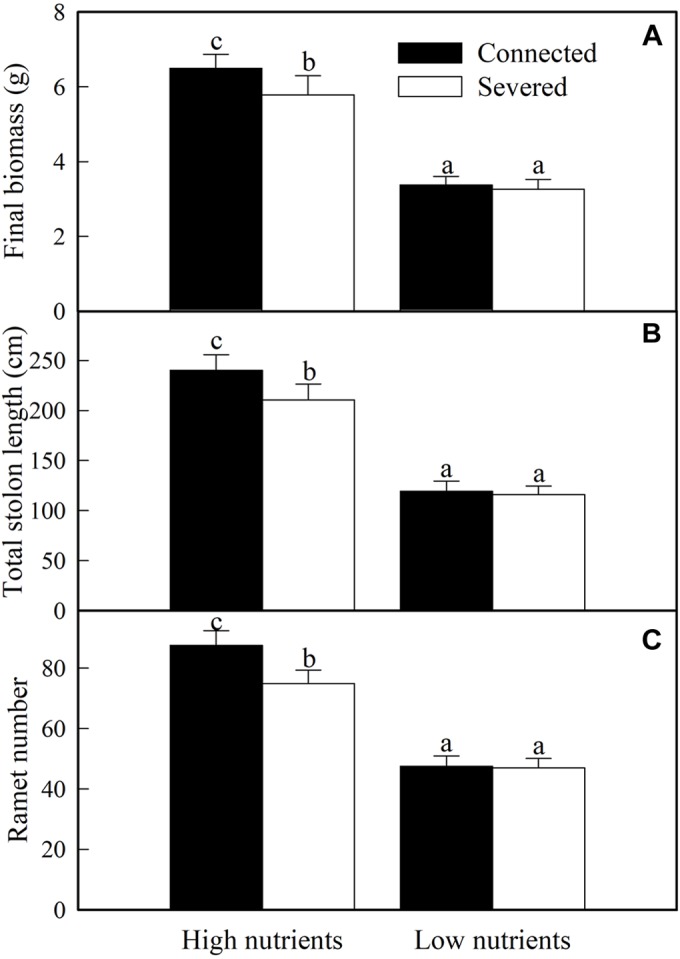
Effects of experimental treatments on the final biomass **(A)**, total stolon length **(B)**, and ramet number **(C)** of the whole clonal fragment of *A. philoxeroide*. The data indicate the means + SE (*n* = 8). The bars sharing the same letter are not significantly different at *P* = 0.05.

The growth of whole clonal fragments (basal + apical ramets) were significantly influenced by soil nutrient, stolon connection and soil nutrient × stolon connection (Table 2). The growth of whole fragments were greatly enhanced by high nutrient supply (Figure 4). When grown in low soil nutrient, stolon connection had no significant effect on the growth of clonal fragments (Figure 4), however, stolon connection significantly increased plant growth when clonal fragments were grown with high soil nutrient supply (Figure 4).

**Table 2:** Two-way ANOVA analyses for the effects of soil nutrient and stolon connection on the chlorophyll content index and chlorophyll fluorescence (yield and *F*_v_/*F*_m_) of *Alternanthera philoxeroides* for the basal part, apical part, and whole clonal fragment.

Dependent variable	Nutrient (N)	Connection (C)	N × C
**Basal**			
Chlorophyll content index	14.72^**^	2.49	1.53^*^
Yield	35.83^***^	2.08	0.10
*F*_v_/*F*_m_	87.00^***^	0.45	0.09
**Apical**			
Chlorophyll content index	16.82^***^	5.45^*^	0.30
Yield	65.14^***^	27.60^***^	4.58^*^
*F*_v_/*F*_m_	131.47^***^	5.73^*^	0.41
d.f.	1.28	1.28	1.28


*Values give *F*; significant *P*-values are indicated by ^∗^. ^∗^*P* < 0.05, ^∗∗^*P* < 0.01, and ^∗∗∗^*P* < 0.001.*

### Chlorophyll Fluorescence and Content

**FIGURE 5:**
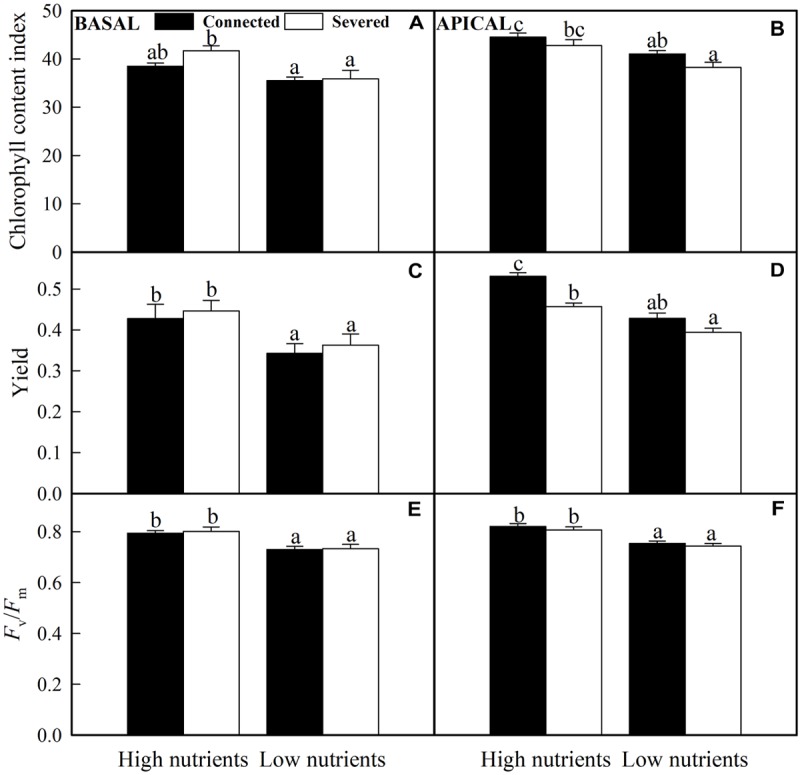
Effects of experimental treatments on the chlorophyll content index, the effective quantum yield of PS II (Yield) and the maximum quantum yield of PSII (*F*_v_/*F*_m_) of basal **(A,C,E)** and apical **(B,D,F)** ramets of *A. philoxeroide*. The data indicate the means + SE (*n* = 8). The bars sharing the same letter are not significantly different at *P* = 0.05.

The chlorophyll fluorescence measurements (Yield and *F*_v_/*F*_m_) and chlorophyll content index of basal ramets were only significantly affected by nutrient treatment (nutrient × stolon connection effect on the chlorophyll content index was also significant), whereas these values of apical ramets were significantly influenced by nutrient and stolon connection (Table 2). High nutrient treatment greatly improved plant chlorophyll fluorescence and content for both basal and apical ramets (Figure 5). However, stolon connection and its interaction with nutrient treatment had no significant effect on chlorophyll performance of basal ramets (Figure 5A,C,E). For the apical ramets, stolon connection had no significant effect on chlorophyll fluorescence and content in low soil nutrient, whereas it greatly enhanced the Yield of *A. philoxeroides* grown in high soil nutrient (Figure 5B,D,F).

## Discussion

As hypothesized, the specialization of basal ramets in root production and apical ramets in shoot production and photosynthetic performance (as estimated by the Yield) were observed within the clonal fragment, supporting the occurrence of the division of labor for *A. philoxeroides* in homogeneous habitats at both morphological and physiological levels. Moreover, we found that the connected apical younger ramets invested more biomass to aboveground structures, resulting in a stronger clonal propagation (estimated by total stolon length and ramet number) and thus greater lateral expansion in both high and low nutrient conditions (Roiloa et al., 2013; You et al., 2016a). These findings agree with those obtained in previous studies on several invasive clonal plants, including *A. philoxeroides* (Wang et al., 2008; You et al., 2014b, 2016a, 2018; Wang P. et al., 2017), *Myriophyllum aquaticum* (You et al., 2013), and *Eichhornia crassipes* (Lyu et al., 2016), which demonstrated that clonal integration can improve clonal propagation and performance of younger ramets and thus allow them to occupy surrounding new space in natural habitats.

As predicted, the division of labor for *A. philoxeroides* was greater in the high nutrient habitat than in the low nutrient habitat (stolon connection increased R/S ratio of the basal ramets more, whereas decreased R/S ratio of apical ramets more, under high soil nutrient conditions than under low soil nutrient conditions), which brought higher clonal propagation (in terms of total stolon length and ramet number) of younger ramets grown in high soil nutrient. This is probably because there was little resource to share for the connected ramets under low nutrient availability, and the division of labor may be relatively weak in such low nutrient conditions (You et al., 2016b). We even detected that the effect of stolon connection on R/S ratio of apical ramets in low nutrient treatment was only marginally significant, suggesting that the factor most limiting the growth of the connected clonal fragments under low soil nutrient conditions might be soil nutrients but not light or lateral expansion (You et al., 2016b). When grown with high nutrient supply, considering that the connected ramets within the fragment are in different developmental stages and thus with different resource storage in the stolons and internodes (Song et al., 2014), and differing in ability to acquire resources (e.g., basal ramets are relatively older with more abundant established roots, whereas apical ramets are relatively younger with less root production), basal older ramets specializing in the acquisition of soil-based resources (such as nutrient and water) in the high-resource condition were considered to be more economical and effective, and apical younger ramets specializing in acquisition of aboveground expansion can facilitate the invasion of clonal invader (Roiloa et al., 2013; You et al., 2016a). Similarly, Wang P. et al. (2017) also detected that clonal integration can increase the new node production and growth of younger ramets of *A. philoxeroides* in homogeneous habitats, especially with higher nitrogen supply, suggesting that clonal integration (division of labor allowed) should be crucial for lateral expansion of this plant invader in some homogeneous habitats (You et al., 2016a).

Although stolon connection allowing the division of labor had positive effects on the performance of apical ramets in both high and low nutrient treatments, such positive effects were generally at the expense of the performance decrease for basal ramets (only the final biomass of basal ramets under high nutrients was not significantly decreased by stolon connection). As a result, stolon connection had different effects on the growth performance of the whole clonal fragments in different soil nutrient conditions. In the low nutrient condition, clonal integration had no significant effect on the growth performance of clonal fragments. This result agreed with Wang P. et al. (2017), who found that clonal integration did not affect clonal performance of *A. philoxeroides* at clonal fragment level in N-limited homogeneous environments. However, clonal integration greatly increased the growth and clonal propagation of clonal fragments in the high nutrient condition. These results occurred most likely because, the capacity for the division of labor of *A. philoxeroides* was greater, which brought it higher benefits at the clonal fragment level in the high nutrient condition than in the low nutrient condition (Dong et al., 2015; You et al., 2016b). Our findings provide strong evidence to support the conceptual model proposed by Dong et al. (2015), which demonstrated that physiological integration (division of labor allowed) may also have a positive effect on the performance of *A. philoxeroides* in homogeneous environments with high resource supply, suggesting that the division of labor may be more important for the invasion of *A. philoxeroides* in homogeneous habitats with high resource supply than with low resource availability (Dong et al., 2015; You et al., 2016b). This may explain in part why *A. philoxeroides* spreads so fast in high-nutrient environments. Therefore, developmentally programmed division of labor may play an important role in determining the invasion of this plant invader to some relatively homogeneous habitats (such as anthropogenic habitats and aquatic ecosystems) (You et al., 2016b).

In heterogeneous habitats, the benefits of the division of labor have been extensively investigated in clonal plants (including some invasive plants) (Stuefer, 1998; Pennings and Callaway, 2000; Wang et al., 2008; You et al., 2013; Roiloa et al., 2016; Lin et al., 2018). However, the present study reported the division of labor for an invasive clonal plant in the homogeneous environment, with all basal and apical ramets subjected to the same external resources. Similarly, Roiloa et al. (2013) also found functional and structural specialization in a stoloniferous clonal plant invader, which benefited from developmentally programmed division of labor in terms of increasing aboveground growth of apical ramets and effective colonization of the surrounding area. The present study provided another strong evidence that the invasive clonal plant can develop the capacity for the division of labor, facilitating its expansion and thus invasiveness under homogeneous conditions. This finding is of great importance, because it proves that the benefits of the division of labor are not only widely existing in heterogeneous environments (Pennings and Callaway, 2000; Saitoh et al., 2002; Wang et al., 2008, 2011; You et al., 2013; Roiloa et al., 2016), but also relevant in homogeneous habitats (Stuefer, 1998; Roiloa et al., 2013). This developmentally programmed division of labor is inherent in clonal plants and irrelevant to environmental heterogeneity (Roiloa et al., 2013). Therefore, besides the relatively low biodiversity, developmentally programmed division of labor may be an alternative mechanism accounted for high invasibility by exotic clonal plants in some homogenized fine-scale conditions such as shallow wetlands and some anthropogenic habitats (You et al., 2016a).

Interestingly, we found that apical ramets always grew better than basal ramets. Such size uniformity might facilitate the division of labor. One possibility may be that clonal integration usually tended to increase the biomass more for the younger, apical ramets to facilitate the lateral expansion, as we mentioned above (Wang et al., 2008; You et al., 2016a; Wang P. et al., 2017). Another possibility may be that the apical ramets had the stolon apexes, which may potentially produce new ramets (Julien et al., 1995). We excluded the possibility that the apical dominance induced the size uniformity, because the original ramets were relatively independent new ramets (the two original ramets of both apical and basal part already rooted before the severing treatment) and probably unlikely affected by apical dominance. However, severance of the apex may affect the growth performance and clonal propagation of the youngest apical ramets, thus additional researches are needed to test this.

More interestingly, besides the benefits of the division of labor detected in the growth and clonal propagation of apical ramets, our results showed that photosynthetic efficiency (estimated by the Yield) of apical ramets was significantly increased by stolon connection, especially in high soil nutrient habitats. This result proved that the division of labor can also be detected at physiological level (Roiloa et al., 2007; Liu et al., 2008; Xu et al., 2010; You et al., 2016b). Recently, we also found a similar benefit of physiological integration (division of labor allowed) in photosynthetic efficiency of *A. philoxeroides* in homogeneous habitats with high water supply (You et al., 2016b). In the present study, the benefits of the division of labor in photosynthetic efficiency were transferred into improved growth and clonal propagation. Owing to such benefits at both physiological and morphological levels, the division of labor may facilitate the colonization capacity and invasion ability, especially in resource-rich habitats (Roiloa et al., 2013; You et al., 2016b).

In short, using a control experiment, we showed that *A. philoxeroides* can develop developmentally programmed division of labor at both physiological and morphological level, thus enhancing its photosynthetic efficiency and growth performance in high soil nutrient conditions. Such benefits of the division of labor could be important for *A. philoxeroides* to colonize new space and spread (You et al., 2016b). In some homogenized fine-scale conditions such as shallow wetlands and anthropogenic habitats, developmentally programmed division of labor may facilitate the invasiveness of exotic clonal plants, which may be an alternative mechanism accounted for notorious plant invasion in these habitats. Furthermore, this study supports the proposal that key clonal traits such as physiological integration and the division of labor can lead to invasion of exotic clonal plants (Song et al., 2013; You et al., 2013). Therefore, prior to introducing alien clonal plants, clonal traits could be important components that could be used to assess their potential invasiveness (Gordon et al., 2012; Song et al., 2013). However, to allow for a comprehensive extrapolation, future in-depth researches are urgently needed on the capacity for the division of labor between invasive and related native plants, or between invasive and non-invasive alien plants (van Kleunen et al., 2010; Wang Y.J. et al., 2017).

## Author Contributions

W-HY conceived and designed the experiments. D-GX, A-AH, and W-HY performed the experiments. D-GX, PH, and W-HY analyzed the data. W-HY and D-LD contributed reagents, materials, and analysis tools. D-GX and W-HY wrote the manuscript.

## Conflict of Interest Statement

The authors declare that the research was conducted in the absence of any commercial or financial relationships that could be construed as a potential conflict of interest.
